# A Unified Computational Framework for a Robust, Reliable, and Reproducible Identification of Novel miRNAs From the RNA Sequencing Data

**DOI:** 10.3389/fbinf.2022.842051

**Published:** 2022-07-08

**Authors:** Vivek Ruhela, Anubha Gupta, K. Sriram, Gaurav Ahuja, Gurvinder Kaur, Ritu Gupta

**Affiliations:** ^1^ Department of Computational Biology & Centre for Computational Biology, Indraprastha Institute of Information Technology-Delhi (IIIT-D), New Delhi, India; ^2^ SBILab, Department of ECE & Centre of Excellence in Healthcare, Indraprastha Institute of Information Technology-Delhi (IIIT-D), New Delhi, India; ^3^ Laboratory Oncology Unit, IRCH, All India Institute of Medical Sciences (AIIMS), New Delhi, India

**Keywords:** miRNA, synthetic RNA-seq data generation, next generation sequencing data, piRNA, paralogues, novel miRNA, RNA-seq

## Abstract

In eukaryotic cells, miRNAs regulate a plethora of cellular functionalities ranging from cellular metabolisms, and development to the regulation of biological networks and pathways, both under homeostatic and pathological states like cancer.Despite their immense importance as key regulators of cellular processes, accurate and reliable estimation of miRNAs using Next Generation Sequencing is challenging, largely due to the limited availability of robust computational tools/methods/pipelines. Here, we introduce miRPipe, an end-to-end computational framework for the identification, characterization, and expression estimation of small RNAs, including the known and novel miRNAs and previously annotated pi-RNAs from small-RNA sequencing profiles. Our workflow detects unique novel miRNAs by incorporating the sequence information of seed and non-seed regions, concomitant with clustering analysis. This approach allows reliable and reproducible detection of unique novel miRNAs and functionally same miRNAs (paralogues). We validated the performance of miRPipe with the available state-of-the-art pipelines using both synthetic datasets generated using the newly developed miRSim tool and three cancer datasets (Chronic Lymphocytic Leukemia, Lung cancer, and breast cancer). In the experiment over the synthetic dataset, miRPipe is observed to outperform the existing state-of-the-art pipelines (accuracy: 95.23% and *F*_1_-score: 94.17%). Analysis on all the three cancer datasets shows that miRPipe is able to extract more number of known dysregulated miRNAs or piRNAs from the datasets as compared to the existing pipelines.

## 1 Introduction

The advent and deployment of high-throughput sequencing techniques triggered the urgent need for the development of computational methods and tools that can robustly, reliably, and reproducibly identify the sequenced molecular identity. To date, a large number of computational methods have been developed for the systematic analysis of these ultra-complex sequencing datasets. However, so far, no single method could robustly achieve the required detection/estimation precision. In pursuit of these, multiple methods are being developed, tested, and deployed. But to date, either the availability as an open-source tool or the anticipated accuracy of their functionality is largely limited.

Reliable sequencing of short RNAs, an essential cellular component known to regulate a plethora of cellular functions, is the much-anticipated breakthrough in sequencing technology. Among these miRNAs, a class of small non-coding RNAs comprising of 17–24 nucleotides has been implicated in regulating transcript homeostasis via RNA degradation pathways (Etheridge et al., 2011; Chevillet et al., 2014; Wang et al., 2016; Kai et al., 2018).

The underlying molecular mechanisms by which miRNAs mature and silence their target transcripts have been extensively studied. However, importantly, due to their centralized position in regulating key cellular processes, a thorough understanding of their identity and hence, their function, both under the homeostatic as well as pathological state is an ever daunting task due to the limited availability of computational methods for their reliable detection. Likewise, in cancer, microRNAs (miRNAs or miRs) have been centrally classified in the networks of oncogenes and tumor suppressor genes (Lin and He, 2017) and therefore, reported to influence diverse transcripts with distinct functions. Loss of function-related experiments in cancer cells pinpointed the exact underlying mechanistic pathways by virtue of which miRNAs regulate cancer initiation and progression. Moreover, due to specific expression of miRNAs in cancer, a large number of them have been proposed as potential biomarkers for cancer detection. Despite their immense importance, reliable computational methods are required for the systematic identification of the novel miRNAs and reliable estimation of their expression levels. Although several methods have been proposed in the past decade for the detection of known and novel miRNAs from the sequencing data, differences in the data processing pipelines of RNA-seq data lead to varying results on the same dataset.

Some of the state-of-the-art pipelines are miRDeep2 (Friedländer et al., 2012), miRDeep* (An et al., 2013), mirPRo (Shi J. et al., 2015), mirnovo (Vitsios et al., 2017), miRge2.0 (Lu et al., 2018), sRNAtoolbox (Aparicio-Puerta et al., 2019), and MiR&moRe2 (Gaffo et al., 2020). The above pipelines for the analysis of smRNAs (small RNAs) yield multiple false positives, do not identify paralogues of existing miRNAs, and often fail to identify the reverse complement sequences of known miRNAs. Although the above methods can detect a number of dysregulated miRNAs, it is important to detect statistically significantly dysregulated miRNAs. These results generally vary across methods because of the algorithm used for extracting the miRNAs along with their count values. Hence, there is a need to develop robust methods to detect accurate and statistically significant dysregulated miRNAs and their count values.

To overcome the aforementioned limitations, we propose miRPipe, a robust computational workflow for the reliable identification and expression estimation of known and novel miRNAs from RNA-seq data. We have performed a comparative analysis of miRPipe with other well-known methods and found that miRPipe outperformed all these methods when benchmarked with both synthetic (known ground truth), and CLL real RNA-Seq expression dataset. To benchmark miRNA pipelines, presently no synthetic data simulators available to generate ground truth. Therefore, we have also developed a highly flexible, innovative, and faster synthetic sequence simulator tool *miRSim* for the comparative analysis of various existing pipelines with miRPipe. Our analysis of CLL datasets identified 31 known and 8 novel dysregulated miRNAs, which we have experimentally validated using real-time PCR on clinical samples. A free friendly synthetic data simulator miRSim and a free dockerized version of miRPipe are available for deployment in a clinical setup. Our aim to provide docker is that our pipeline miRPipe is easily usable for both beginner and expert bioinformaticians. They can subsequently share the analyzed results with the clinicians for further inference.

## 2 Materials and Methods

We have used synthetic and real RNA-Seq expression datasets for functionality and benchmark against the available state-of-the-art miRNA pipelines. Of note, we have developed an in-house tool miRSim (Ruhela et al., 2021) to assess the pipeline performance by comparing pipeline outcome with matched ground truth. For miRPipe validation, we have considered three publicly available GEO datasets, that is, Chronic Lymphocytic Leukemia (CLL) dataset (GSE123436) (Kaur et al., 2020), breast cancer dataset (GSE171282) (Lin et al., 2021) for miRNA identification and Lung Cancer dataset (GSE37764) (Nogueira Jorge et al., 2017) for piRNA identification.

### 2.1 Proposed miRSim Tool: Synthetic Data Simulator

The performance assessment of existing or the development of any new bioinformatics tool such as a sequence aligner or a sequence quantification tool is robustly possible only when the ground truth is available. Presently, some of the synthetic sequence simulators available for the generation of synthetic sequencing data such as ART (Huang et al., 2012), pIRS (Hu et al., 2012), Flux (Griebel et al., 2012), polyester (Frazee et al., 2015), and RNA-Seq simulator (Grant et al., 2011), can also be used to generate reads containing miRNA sequences. These synthetic RNA-Seq data generator tools are generic in nature, and data generation depends on platform-specific parameters. However, these tools do not provide the ground truth data for the validation of a bioinformatics pipeline. Hence, we designed the miRSim tool that fills this gap.

The design and workflow outline of the miRSim tool is illustrated in 1. Mechanistically, the standard miRNA sequences and their genomic location can be stored in FASTA and GFF file formats (gff3) as the reference input to the miRSim tool. The miRNA, piRNA, and novel miRNAs sequences were collected from the miRBase (Kozomara et al., 2019), piRNAdb database (version 1.7.6) (https://www.pirnadb.org/), and the recent literature (Wallaert et al., 2017; Kaur et al., 2020), respectively. For the robustness of any RNA-Seq pipeline, it is essential to detect known miRNAs, novel miRNAs, and their paralogues robustly. Hence, miRSim provides the option to add a selected percentage of altered sequences of miRNAs and piRNAs as the ‘Other’ category, which acts as a true negative to assess the efficiency of the pipeline.

**FIGURE 1:**
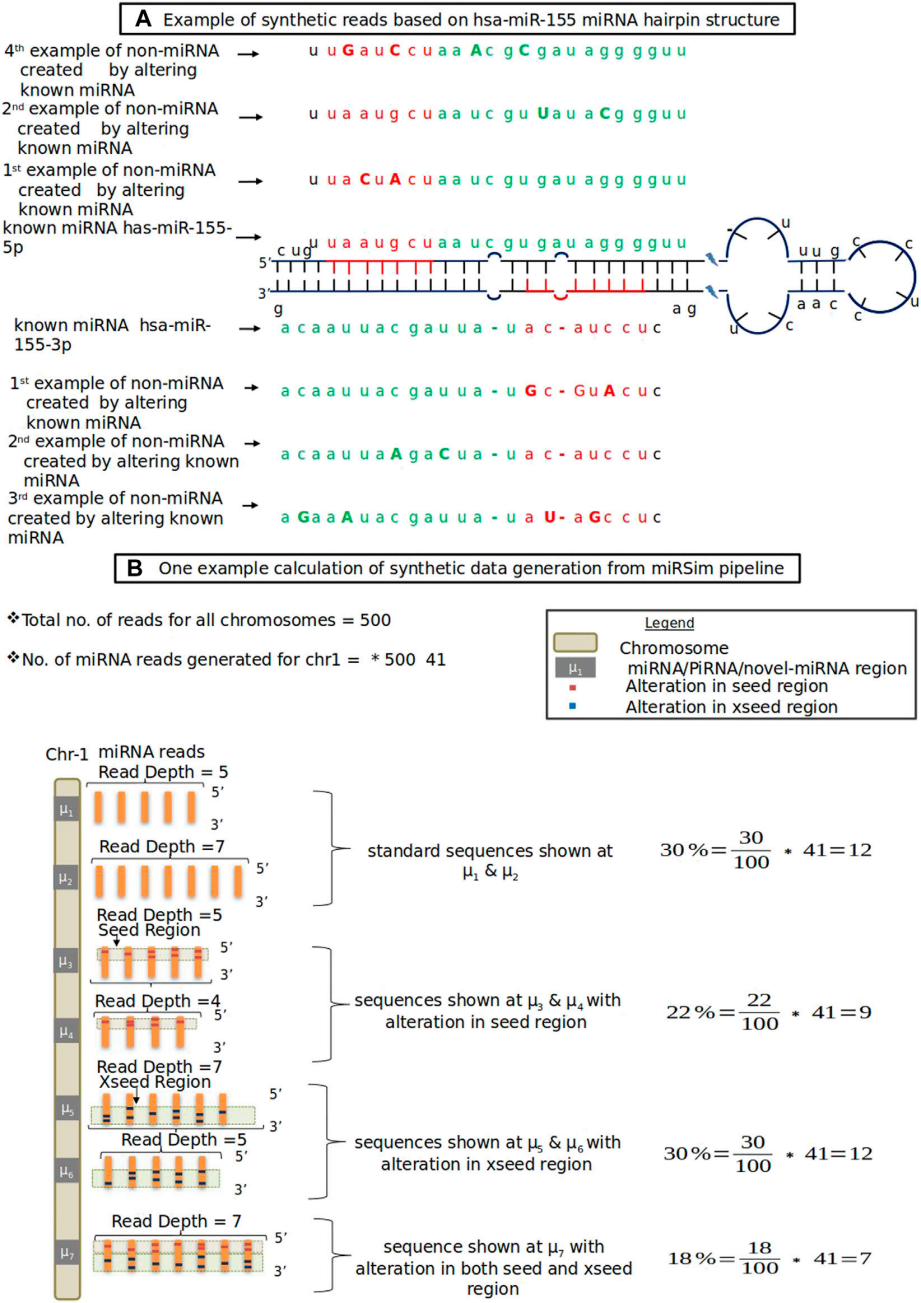
**(A)** Example of synthetic reads based on hsa-miR-155 miRNA hairpin structure. Red color shows seed region, green color shows xseed region and capital letters denote altered nucleotide **(B)** One example data from miRSim pipeline. Here, the miRNA/piRNA region is represented by *μ*_1_, *μ*_2_, …. Here *μ*_1_ represents original miRNA and *μ*_2_-*μ*_7_ are derived from *μ*_1_ by alterations in the seed and xseed sequence of *μ*_1_ and may or may not constitute a valid miRNA. The number of miRNAs present in the chromosome-1 and total number of miRNAs in all chromosomes are taken from miRBase (version22) (Kozomara et al., 2019)

In this category, the new miRNA sequence is generated by altering either the seed region’s nucleotides (red colored nucleotides in Figure 1A) or by altering the xseed region’s nucleotides (green colored nucleotides in Figure 1A) or both by at least 2 nt. The altered nucleotide are shown in each of the seed and xseed regions by capital letters. The seed and xseed regions (regions other than the seed region) of a miRNA govern the functionality of miRNA in biological processes (Kehl et al., 2017). The 2 nt cut-off was based on the fact that the recommended tolerance used in the standard RNA-seq aligners such as STAR (Dobin et al., 2013), TopHat2 (Kim et al., 2013), miRDeep2, and miRDeep* is allowed upto 2 nt. The resulting sequence will not be a miRNA or piRNA. The fraction of sequences for each of these error types is provided in the form of an error profile as input to the miRSim tool by the user. One example is shown in Figure 1. Here, Figure 1A shows the hairpin structure of hsa-mir-155 with highlighted seed (red-colored nucleotides in Figure 1A) and xseed (green colored nucleotides in Figure 1A) regions. Similarly, Figure 1B shows one example calculation of synthetic sequence generation by miRSim by doing alterations in the miRNA sequences.

*Workflow of miRSim Tool:* The miRSim tool accepts reference sequences of miRNAs and their genomic location from the input fasta and gff files provided by the user. In addition, the user provides other input parameters such as the total number of sequences to be generated, % of known miRNAs, % of novel miRNAs, % of known piRNAs, quality score encoding, minimum depth and expression profile distribution for generating the synthetic data. The miRSim tool utilizes this information first to infer the distribution of reads (that is, the number of reads per chromosome). The read distribution is directly proportional to the number of miRNAs present in each chromosome. Using the read distribution, the number of miRNAs per chromosome is computed such that each miRNA gets a read depth greater than or equal to the minimum required read depth. Using this miRNA distribution, each chromosome’s expression profile is generated based on either the Poisson or the gamma distribution. Finally, the synthetic sequences are generated by adding the adaptor sequence and primer sequence such that the overall sequence length becomes 75 and written in the output FASTQ/FASTA file. The miRSim tool supports parallel thread operation to write the synthetic sequences in multiple threads simultaneously in order to generate data expeditiously.

miRSim also provides the ground truth in a readable comma-separated file format that contains information about known miRNAs, piRNAs, and novel miRNAs along with their sequences, chromosome location, expression counts, and the CIGAR string for all the sequences. The ‘Other’ category sequences also specify the known miRNAs/piRNAs (with chromosome location) from which these altered sequence reads are generated besides the above information. Any robust pipeline should discard these noisy reads. miRSim delivers output in the form of a compressed FASTQ or FASTA file format. As of now, miRSim tool has been developed and tested for the human genome only. For other genomes, miRSim parameters can be readjusted accordingly. For other non-human genomes, a user has to adjust 1). RNA-sequence length for that genome, and 2). seed region and xseed region location. The core algorithm will remain the same. The source code of miRSim is available in the GitHub and zenodo in both command line version and jupyter notebook version.

### 2.2 Synthetic RNA-seq Expression Dataset Used in This Study

In this study, we have generated the miRSim simulated synthetic dataset for the benchmarking of the pipeline on the identification of known/novel miRNAs and known miRNAs. Using miRSim, we generated thirty synthetic FASTQ files with a varying read depth of 50 K reads, 0.1 million reads, and 1 million reads with known proportions of both altered and unaltered known/novel miRNAs and known piRNAs (Supplementary Material S6). The reason behind considering the multiple read depth categories is to assess the pipeline for low depth as well as high depth data. Although, the synthetic data experiments can also be extended to higher depth (more than 1 million reads). For known miRNA identification experiments, reads were generated using high confidence miRNAs taken from miRBase (version 22) to ensure the least false positives or false negatives in the experimental design. Similarly, for novel miRNA identification, miRpipe includes the genomic and structural features. Novel miRNAs detected recently in (Wallaert et al., 2017; Kaur et al., 2020) were added to the synthetic data experiments as the ground truth sequences (See Supplementary Material S9). Moreover, for known piRNA identification, the reads were generated from the piRNAdb database (version 1.7.6). We have also generated the synthetic data file for benchmarking pipelines on the identification of reverse complement sequences as known miRNAs. For this purpose, we have generated a synthetic FASTQ file that had the reads of 887 high confidence miRNAs (added from miRBase database version 22) with a read depth of 10 each. Thus, the synthetic FASTQ file contained 8,870 reads with reverse complement of 887 high confidence miRNAs.

### 2.3 Real RNA-seq Expression Datasets Used in This Study

We have included three publicly available datasets in our study that are CLL dataset (GSE123436), breast cancer dataset (GSE171282), and lung cancer dataset (GSE37764) for miRPipe validation. In the CLL dataset, the RNA-Seq profile of 28 CLL cases and 10 age-matched healthy controls were studied to identify unique pattern of eight dysregulated miRNAs in CLL. This study also validated the altered expression levels of eight dysregulated miRNAs by RT-qPCR. The breast cancer dataset (GSE171282) consists of 3 normal and 6 tumor RNA-Seq samples. The breast cancer dataset was studied to understand the effects of two commonly used local anesthetics, lidocaine, and bupivacaine, on the malignancy of MCF-7 breast cancer cells. The original publication of the breast cancer dataset (GSE171282) has reported 11 RT-qPCR validated dysregulated known miRNAs. We have used CLL and breast cancer datasets for the miRPipe validation in miRNA identification. Similarly, in the lung cancer dataset, the primary non-small cell lung adenocarcinoma tissues of 6 never-smoker Korean female patients were studied to identify dysregulated piRNAs to identify the altered expression patterns of non-coding RNAs in the non-smoker females. The original publication of the lung cancer (GSE37764) dataset has not reported any dysregulated piRNAs. We have used this dataset for miRPipe validation in piRNA identification.

### 2.4 Description of the Proposed miRPipe

**FIGURE 2:**
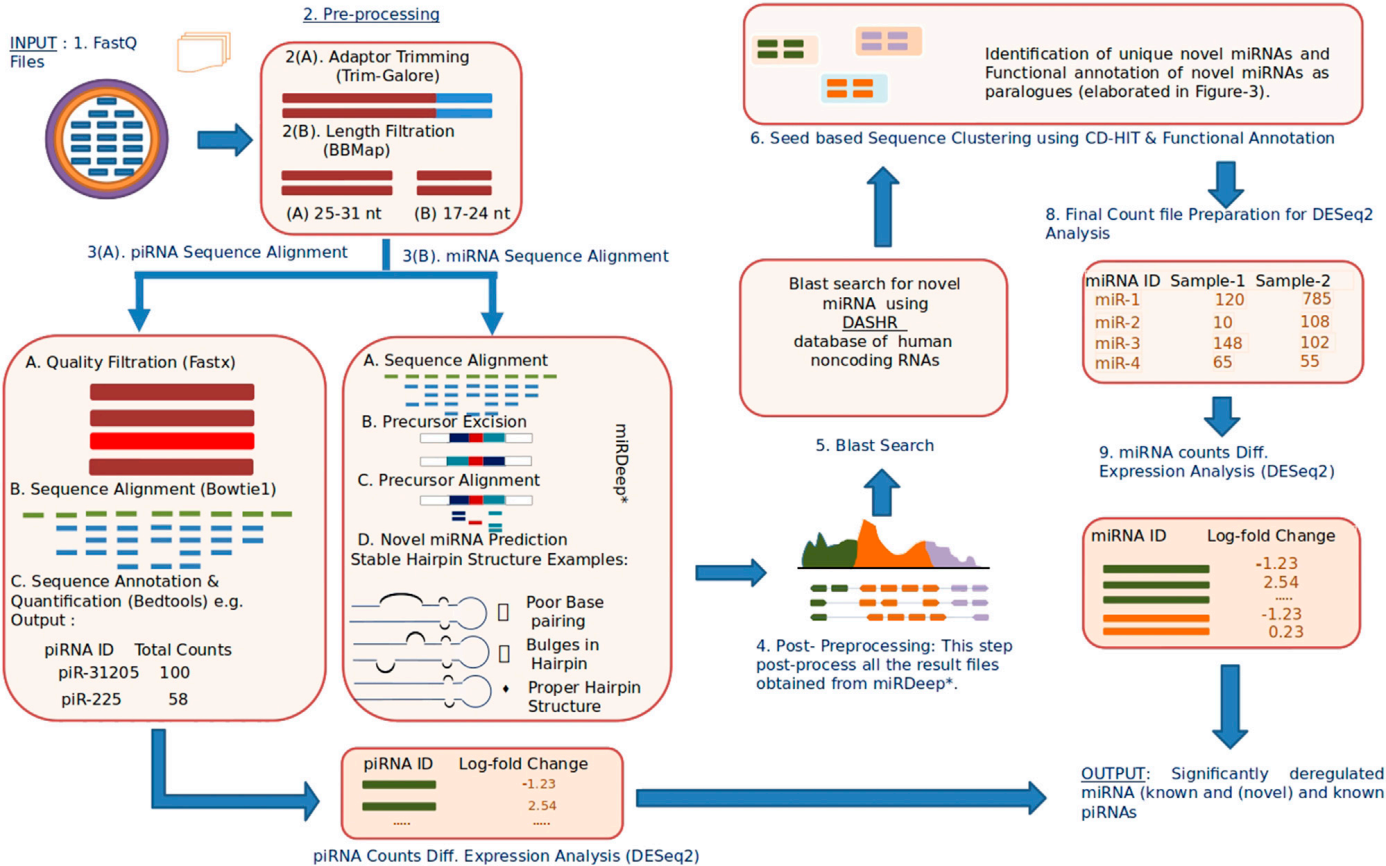
Infographic representation of miRPipe workflow: This pipeline identifies differentially expressed novel miRNAs, known miRNAs, and known piRNAs.The RNA-Seq data in standard FASTQ format is cleaned for adaptor contamination. Reads of specific lengths are aligned to the human reference genome for miRNA and piRNA identification. Further, the aligned reads are processed using novel seed-based clustering for their functional annotation. Lastly, their differential expression analysis is computed using DESeq2 to find the significantly dysregulated miRNAs and piRNAs.

A complete block diagram of miRPipe is provided in Figure 2.

#### 2.4.1 Input Data

The miRPipe allows both single or multiple sample processing with input files in FASTQ format (either.fastq or.fastq.gz. For computing the differentially expressed miRNAs, miRPipe utilizes the widely accepted DESeq2 method. Importantly, the information on adaptor sequence, human reference genome, and miRBase version can be either provided by the user or the default options of miRPipe can be chosen.

#### 2.4.2 Hardware and Software Dependencies

Pipelines: miRPipe, miRDeep* (An et al., 2013), miRDeep2 (Friedländer et al., 2012), mirPRo (Shi J. et al., 2015), sRNAtoolbox (Aparicio-Puerta et al., 2019), miRge2.0 (Lu et al., 2018), mirnovo (Vitsios et al., 2017), MiR&moRe2 (Gaffo et al., 2020) were installed and run on a workstation with the hardware configuration of Single Intel(R) Core(TM) i7-8700 CPU 6Cores, 12Threads,@Base frequency of 3.20GHz, 32 GB DDR4 RAM. The docker is fully functional on Linux platform and requires the following system configuration: Ubuntu 18.04 operating system with at least 8 GB RAM. The miRSim tool was developed on hardware configuration of Single Intel(R) Core(TM) i5-8400 CPU 2Cores, 4Threads, @Base frequency of 2.80GHz, 8 GB DDR4 RAM.

### 2.5 miRPipe Workflow

miRPipe is an integrated pipeline for the identification of statistically significant differentially expressed known/novel miRNAs, and known piRNAs simultaneously by parallel threaded operations.

**FIGURE 3:**
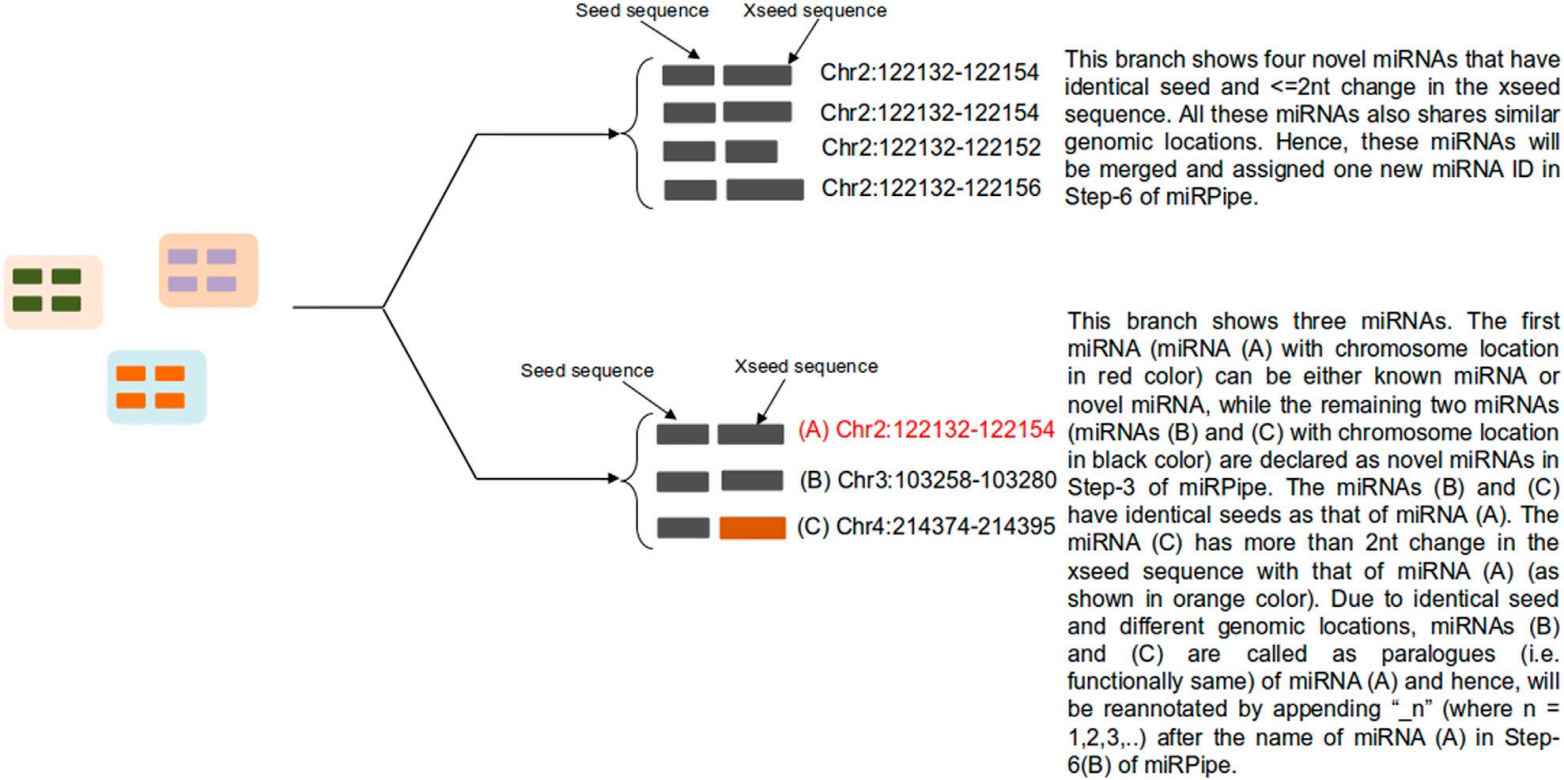
Functional annotation of novel miRNAs using seed-based clustering. Above figure shows an example for all possible scenarios for cluster formation of novel miRNAs functional annotation with known and novel miRNAs along with their genomic location of sequence alignment.

The following steps are sequentially carried out in the miRPipe (Figure 2):

1) *Input:* miRPipe accepts sequencing files in the FASTQ format, along with the sample information file in the CSV format that contains sample (or subject) IDs and sample group (whether treated and control, or the data collected at two different time points).
2) *Pre-processing:* miRPipe performs adapter removal in the raw FASTQ files using the Trim-Galore tool (http://www.bioinformatics.babraham.ac.uk/projects/trim_galore/). Post-trimming, miRPipe segregates reads based on their sequence lengths. The first category contains read sequences of 17-24 nt lengths that are processed further for miRNA identification.The second category contains read sequences of 25-31 nt lengths that are processed for piRNA identification. The remaining read sequences of lengths ≥32 nt are rejected by miRPipe.
3) *Sequence Alignment:* In Step-3, miRPipe initializes parallel threads for 1) the identification of known and novel miRNAs and 2) the identification of piRNAs. While one CPU thread is allocated for piRNA identification, the remaining CPU threads are allocated for miRNA identification.
  3A) *piRNA Identification Pipeline:* For piRNA identification, reads of length 25–31 nt are screened based on their quality scores. Reads having more than 10% bases with a phred score of less than 20 are filtered out. The remaining reads of better quality are aligned to human genome using the bowtie (version 1.2.3) with the following fixed parameters: 1) minimum length of sequence *l* = 25; 2) zero mismatch in the seed region; 3) with no reverse complement alignment allowed to obtain the alignment results. All the alignment results are then post-processed using the bedtools intersect. miRPipe utilizes piRNA annotations from piRNAdb. Subsequent analysis results in a final count matrix of all the annotated piRNAs across all samples that are used as input for the DESeq2 for the differential gene expression analysis.
  3B) *miRNA Alignment*: The first step in miRNA identification is the sequence alignment using miRDeep*. miRDeep* sequence aligner is developed on the top of bowtie and allows the sequence mapping with zero mismatches in both strands of the human genome reference.
4) *Post-Processing*: All known miRNA and novel miRNA reads are collected from all the samples (multiple subjects) to prepare a list of reads for the DASHR blast search processing.
5) *Blast search using DASHR*: All miRNAs that are not annotated as known miRNA are blast-searched with the DASHR database to check if they match with any known miRNA sequences. Moreover, there can be some sequences that are annotated as novel miRNAs, whose annotation is missed due to it being present as a reverse complement sequence in the fastq file. Although bowtie can map a reverse complement sequence of a known miRNA to its correct genomic location, yet due to differences in the mapping strand and precursor sequence from the respective mapping strand and precursor sequence of that known miRNA, miRDeep* fails to annotate the reverse complement sequence to its true known miRNA. Such cases are referred as a novel by miRDeep*, although they should have been assigned as known miRNA. Thus, in such cases, we blast search these sequences in the DASHR database and check if they match with any of the known miRNAs. The DASHR database tries to find the best possible match with known miRNAs (according to the reference genome hg19 or hg38 as specified by the user). If the DASHR database does not find any hit with any known miRNA, then we take the reverse complement of these sequences and blast search again in the DASHR database. Now, if they match any known miRNA at the same genomic location as that of novel miRNA, the novel miRNA will be re-annotated as known miRNA, and the count of novel miRNA will be merged with that of known miRNA. For other reads, the miRNA nomenclature system used in miRBase (Ambros et al., 2003) is followed for their renaming as explained in the next step.
6) *Novel Seed-based clustering and Functional annotation of novel miRNAs:* In Step-6, miRPipe performs the seed-based clustering on both known and novel miRNA sequences. The methodology of seed-based sequence clustering and functional annotation is as follows:
  6A) *Novel Seed-based clustering:* In this step, seed-based clustering is employed by miRPipe on known and novel miRNAs to identify unique novel miRNAs and known miRNA paralogues. Different scenarios of seed-based clustering is shown in Figure 3. First, we perform CD-hit (Li and Godzik, 2006) clustering on the seed sequences of all novel and known miRNAs. novel miRNAs whose seed sequences are identical (that is, 0 nt mismatch) are subsequently checked, again via CD-hit clustering, but now on xseed region sequences. All novel miRNA reads having identical seed sequence, maximum alterations of 2 nt in the xseed sequence, and similar genomic location (that is, maximum 2 nt variation in the genomic position) were identified as a single novel miRNA. Their counts were merged.
  1B) *Functional annotation of novel miRNAs:* According to (Bofill-De Ros et al., 2019), if a given sequence has the identical seed sequence with different genomic location, such a sequence are called as the paralogous. Using these criteria, all novel miRNAs that shares the identical seed with different genomic location are called as paralogues. If the novel miRNA has the identical seed as that of a known miRNA (say hsa-mir-x), and a different genomic location, then the novel miRNA will be called as paralogue of known miRNA and will be labeled as “hsa-mir-x_n” where n is 1,2,3,…as more paralogues are discovered. Similarly, if the novel miRNA has the identical seed as that of a novel miRNA (say novel-mir-x) and a different genomic location, then the novel miRNA will be called as paralogue of novel miRNA and will be labeled as “novel-mir-x_n” where n is 1,2,3,…as more paralogues are discovered. Functionally, the paralogues behave identically (Begley and Ellis, 2012) due to the same seed in their mature miRNA sequence.
7) *Final count file preparation*: Once the functional annotation of novel miRNAs is completed, miRPipe returns the final count matrix containing expression counts of all novel miRNAs, known miRNAs, and known piRNAs across all the samples. Since there are many miRNAs in miRBase whose mature sequences are identical and located at multiple genomic locations in the human genome. Such miRNAs represent the miRNA paralogues. The sequence aligner in Step-3 of miRPipe workflow lists all these miRNA paralogues with the same expression counts. Thus, in real RNA-Seq expression data, miRPipe deduplicate the final count file to remove the multiple entries of the same mature sequence present in the count file.
8) *Differential Expression Analysis*: miRPipe carries out DEM (dysregulated miRNAs) analysis using the DESeq2 method. Any miRNA or piRNA is considered to be statistically differentially expressed if its p-adj value after Benjamini–Hochberg (BH) correction is ≤0.05.
9) *Renaming of novel miRNAs*: The dysregulated novel miRNAs identified in Step-8 of miRPipe workflow are renamed using the miRNA nomenclature system used in miRBase [(27), http://www.mirbase.org/help/nomenclature.shtml]. The rules for miRNA nomenclature are as follows:
  a) *Previously annotated miRNAs:* If the novel miRNA sequence has been already annotated in another organism, then the same identifier will be used in other organisms also. For this, we have blast searched all the novel miRNA sequences in the Rfam database with E-value less than or equal to 0.01 and then renamed them using the same identifier.
  b) *Sequential annotation:* If the above conditions are not met for any novel miRNAs, then the renaming was done sequentially. In the end, we have added “*” in the suffix of all novel miRNA new names to represent that these names are putative names only.
10) *Output*: The final output file in the corresponding user data directory contains the significantly differentially expressed miRNAs and piRNAs.

## 3 Results

### 3.1 Benchmarking of miRPipe With Existing Standard Pipelines

miRPipe is benchmarked with seven standard pipelines introduced in the recent past for the novel miRNA detection. These are mirdeep2, mirdeep*, mirPRo, mirnovo, miRge2.0, sRNAtoolbox, and MiR&moRe2. We have proposed an innovative strategy for miRNA pipeline validation and benchmarking, where we have used synthetic RNA-Seq data with known ground truth and real RNA-Seq expression data. The synthetic data experiments allow us to evaluate the accuracy, sensitivity, and specificity for extensive comparison with other pipelines in identifying and annotating correct miRNAs. Hence, results are presented: 1) by comparing the workflow and architecture of all the pipelines, 2) by using the pipelines on the synthetic data generated by the miRSim tool, where the ground truth is known, and 3) by comparing the results obtained on the real RNA-Seq expression CLL, lung cancer and breast cancer dataset.

### 3.2 Summary Comparison of Different Pipelines

We have compared the bioinformatics pipeline parameters (such as sequence quality control, miRNA sequence length criteria, sequence aligner, type of genome dataset used, reference annotation database, type of RNA considered, etc.), and features (such as miRNA profiles, model types, platform dependencies, etc.) of all the eight pipelines and shown in Table 1. The sequence pre-processing in any bioinformatics pipeline includes three sequence filtering/trimming steps, that is, 1. adaptor-trimming, 2. quality control, and 3. length control. The adaptor trimming and quality control steps are mandatory to prepare reads for downstream analysis, such as sequence alignment with respect to the reference genome, miRNA annotation, etc. In the length filtration step, all eight pipelines have different criteria of miRNA sequence length, e.g., miRDeep2 and miRDeep* consider sequence having a length range of 18 nt-23 nt as miRNA. In contrast, miRPro considers all the sequences having a length greater than 17 nt as miRNA, etc. We have observed that most of the human miRNAs lie in the range of 17 nt-24 nt. Also, both miRNAs and piRNAs sequences have slightly varying lengths across different miRNAs and piRNAs instead of strictly defined fixed lengths. For example, there are some known piRNAs whose sequence length is 25, while some others have lengths less than 25. Thus, it is challenging to find the exact cutoff of sequence length that can help infer a maximum number of true positive miRNAs or piRNAs.

**TABLE 1:** Comparison of recently published bioinformatics pipelines on all the intermediate steps such as sequence pre-processing, de-duplication, sequence alignment, and sequence annotation. In all the intermediate steps, each pipeline uses different tools (with different versions) or their own module written in languages such as *Python*, Perl, R or C++. Further, each pipeline has its own miRNA consideration criteria. For example, miRge2.0 pipeline considers 16–25 nt length of sequence for miRNA identification, while sRNAtoolbox considers all the sequence of length less than 25 nt for miRNA identification. This difference in length criteria plays a significant role in the difference in the accuracy of miRNA sequence alignment step, which is the most crucial step in the bioinformatics pipeline for miRNA identification. Majority of the above-mentioned pipelines uses Bowtie1 for sequence alignment, while mirnovo pipeline uses Bowtie2 and mirPRo pipeline uses Novoalign sequence aligner. In our proposed miRPipe, we have used the miRDeep* in Step-3 of the workflow with DASHR blast search and seed-based clustering of the novel miRNAs. Most of the pipelines also report other categories of RNAs present in the sequencing data such as rRNA, moRNA, piRNA, etc. We have also integrated our pipeline piRNA identification pipeline in miRPipe with parallel thread execution for an optimum use of computational resources to facilitate less overall time to deliver the output results.

Pipeline steps	Modules	Pipelines							
		miRDeep2 (Friedländer et al., 2012)	miRDeep* (An et al., 2013)	mirPRo (Shi et al., 2015a)	Mirnovo (Vitsios et al., 2017)	miRge2.0 (Lu et al., 2018)	sRNAbench (Aparicio-Puerta et al., 2019)	MiR&moRe2 (Gaffo et al., 2020)	miRPipe
Year		2012	2013	2015	2017	2018	2019	2020	
Pre-processing	Adaptor Trimming	✓	✓	*✓*	Reaper	Cutadapt (v1.11)	*✓*	Cutadapt (v2.5)	Trim-Galore
	Quality Control (Min Phred Score)	20	20	*✓*	×	20	20	20	20
	Length Control	18–23 nt	18–23 nt	> 17 nt	×	16–25 nt	< 25 nt	15–30 nt	17–24 nt (miRNA) 25–31 nt (piRNA)
Sequence Deduplication	Collapsed to Unique reads	✓	✓	*✓*	Tally + vsearch + CD-HIT	*✓*	*✓*	*✓*	*✓*
Sequence Alignment w.r.t. References Genome	Sequence Alignment	Bowtie (v1.1)	Bowtie (v0.1)	Novoalign + HTSeq	Bowtie2 + mirnovo_analysis.pl module	Bowtie (v1.1.1) + Samtools	Bowtie (v0.12)	Bowtie (v1.1.2) + Samtools + bedtools (v2.27)	Bowtie (v1.2.3)
Sequence Annotation	miRNA Database	miRBase	miRBase	miRBase	miRBase	miRBase/miRGeneDB	miRBase	miRBase	miRBase
	Other RNA Database	×	×	×	Rfam	Ensembl	Provided by user	*✓* (Experimental)	piRNADb
	miRNA Identification	✓	✓	*✓*	*✓*	*✓*	*✓*	*✓*	*✓*
	Isomirs	✓	✓	*✓*	*✓*	*✓*	*✓*	*✓*	*✓*
	novel miRNAs	✓	✓	*✓*	*✓*	*✓*	*✓*	*✓*	*✓*
	Other sncRNA	×	×	×	rRNA, tRNA	Primary tRNA, rRNA, snoRNA, known RNA spike-in sequence	tRNA, snoRNA, snRNA, rRNA, piRNA	moRNA, loopRNA	piRNA
Features	Genomic features	×	×	×	9	×	×	×	×
	Coverage Profile features	×	×	×	12	×	×	×	×
	Sequence Features	✓	✓	*✓*	12	21	*✓*	*✓*	*✓*
Model Type	Machine Learning based	No	No	No	Random Forest	Support Vector Machine	Weka	No	No
Application Genome	Human	✓	✓	*✓*	*✓*	*✓*	*✓*	*✓*	*✓*
	Non-human	✓	✓	Mouse, Chicken	8 animal species and 7 plant species	novel miRNA prediction only for Mouse genome	*✓*	×	×
Language	Programming Language	Perl, Bash	Java	C++	Perl, R	*Python* 2.7	Web Server	Pytyon3, R Bash	*Python*3, Bash
	Packages				Random Forest R Package	Biopython, Numpy, Scipy, pandas, sklearn, reportlab, forgi python packages		data.table R Packages	
OS Supported		Linux	Linux, MACOSX Windwos	Linux	Linux MACOSX	Linux	Not required	Linux	Linux, MACOSX and Windows

Moreover, there are 1.3% miRNAs that are present in the miRBase database having sequence lengths of 25 nt to 28 nt. At the same time, there are 21.3% piRNAs that are present in the piRNAdb database having sequence lengths of 25 nt to 28 nt. Considering sequences of length 25 nt to 28 nt for miRNAs may lead to false negatives in the piRNAs pipeline, and we may miss many important piRNAs. Similarly, rejecting a sequence of length less than 18 nt may lead to ignoring the actual miRNAs. Hence, we consider the sequence length range of 17 nt-24 nt for miRNAs and 25 nt-31 nt for piRNAs. Thus, all the steps in sequencing data pre-processing, especially length filtration, play a crucial role in pipeline performance evaluation.

In addition, the selection of sequence aligners also plays an important role in sncRNA identification. Most of the pipelines have used bowtie1 except mirPRo and mirnovo because bowtie1 is very sensitive to small and medium-length sequences. For miRNA annotation, miRBase is considered a standard database used in all eight pipelines except miRge2.0, which lets the user choose the annotation database (either miRBase or miRGeneDB) as per user requirement. In addition to identifying known and novel miRNAs, some pipelines like mirnovo, miRge2.0, MiR&moRe2, and sRNAToolbox also provide information about other RNA types such as tRNA, rRNA, snoRNA, moRNA, loop-RNA, etc. Only three out of the seven existing pipelines (mirnovo and miRge2.0, sRNAToolbox) use the random forest, SVM, and Weka-based machine learning model for novel miRNA prediction. Also, each pipeline has been developed using a different programming language and has different platform dependencies. The difference in the miRNA selection criteria, sequence alignment strategy, annotation database, the model used for snc-RNA identification, etc., makes these pipelines methodologically unique and cause different outputs for the same input data.

### 3.3 Benchmarking of Pipelines on Synthetic Data

Synthetic data is generated using miRSim tool for pipeline validation with known percentage of reads of known/novel miRNAs, and known piRNAs (Refer to Supplementary Material S6 for an example). Since the ground truth is known, pipelines are assessed on the metrics of accuracy and *F*_1_-score. The following notations are used for four class label detection: Class-1: known miRNA, class-2: novel miRNA, class-3: known piRNA, class-4: Not belonging to other 3 classes.

• A read is counted as true positive (TP) if it is correctly identified by the pipeline.
• A read is considered as false positive (FP) to class-‘x’, when it belongs to one of the other classes, but is identified as class-‘x’ read.
• A read is considered as false negative (FN) when it belongs to class-‘x’, but is rejected by the pipeline to class-4 (Others).
• A read is considered as true negative (TN) when it is an altered miRNA/piRNA and is also labeled the same by the pipeline, that is, all the reads with sequences not belonging to valid miRNAs or piRNAs or novel miRNAs (in other words, not belonging to any of the above three classes) are called as true negatives.

The performance metrics are computed as:

$$Accuracy=\frac{TP+TN}{TP+TN+FP+FN},$$

$$F_{1}\;\;Score=2.\frac{Precision\times Recall}{Precision+Recall},$$

$$\text{where }Precision=\frac{TP}{TP+FP}\text{ and, }$$

$$Recall=\frac{TP}{TP+FN}.$$

#### 3.3.1 Benchmarking of Pipelines on the Identification of Known miRNAs

**FIGURE 4:**
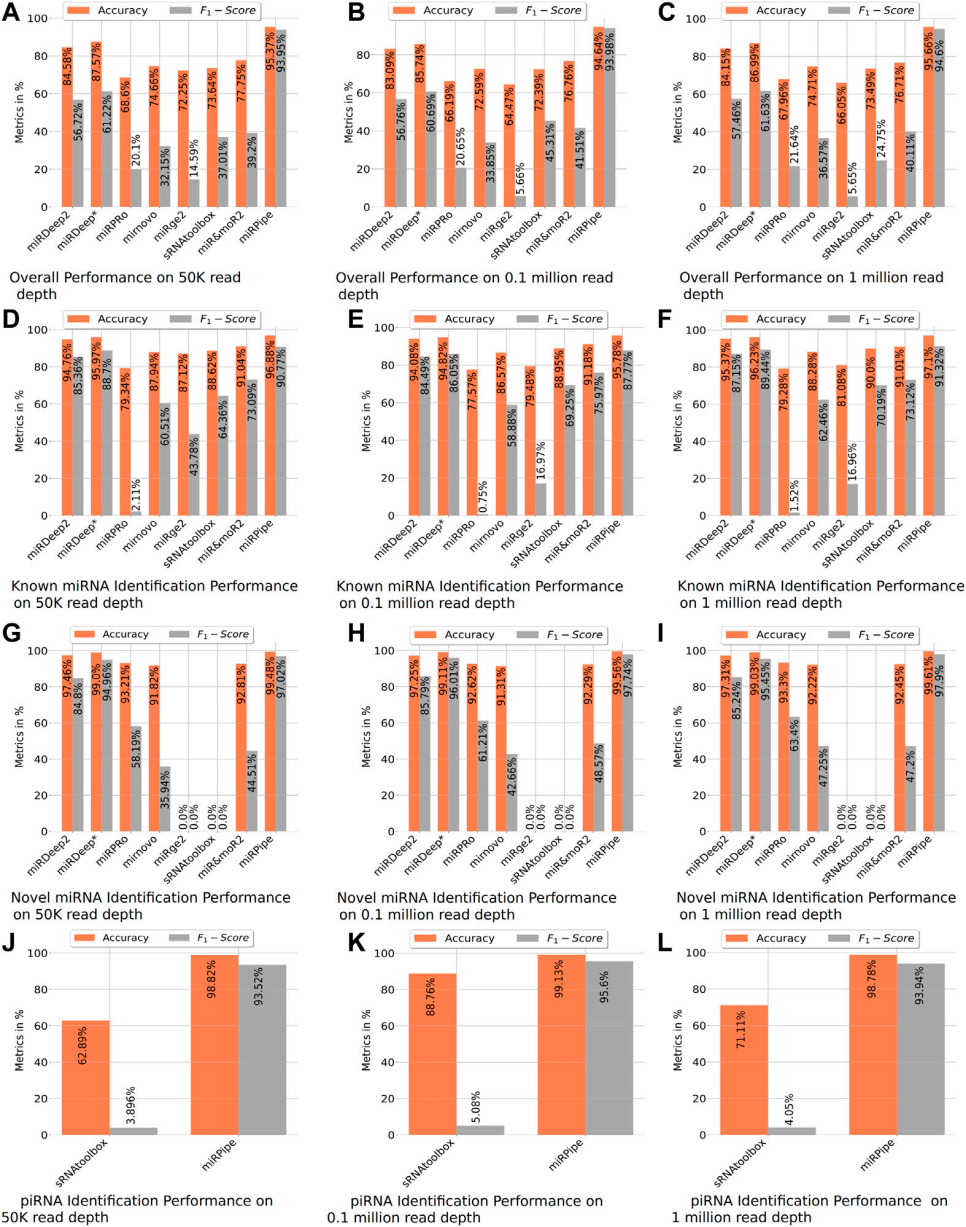
Benchmarking of miRPipe with the existing pipelines on synthetic data. Averaged results are reported over 10 FASTQ files generated for each read depth of 50 k, 0.1 million, and 1 million reads. The overall performance of all existing pipelines are shown in **(A–C)**. All the pipelines are benchmarked against miRPipe for known miRNA in **(D–F)** and for novel miRNAs in **(G–I)**. Among the existing pipelines, only sRNAtoolbox identifies piRNA and hence, comparison results of miRPipe for piRNA are compared with only sRNAtoolbox in **(J–L)**.

In the synthetic data experiment for the identification of known miRNAs, we have observed that there are many mature miRNA sequences that can match at multiple genomic locations on the human genome, wherein the miRNAs at these different genomic locations correspond to different precursor sequences. For such cases, we have compared the miRPipe outcome with the miRSim generated ground truth for miRPipe pipeline assessment. The comparative analysis with miRPipe revealed that miRPipe outperforms existing pipelines with an average accuracy (across all depths) of 96.58% and an average *F*_1_-score of 89.95% on the identification of known miRNAs (Table 2; Figure 4, and Supplementary Material S1–S3, synthetic data experiment results for 50 K, 0.1 and 1 M read depth).

**TABLE 2:** Average performance of pipelines for known miRNA, novel miRNA and known piRNAs. The cells with ‘-’ indicates that pipeline does not identify that particular type of RNA.

Pipelines	Average accuracy Across all Depths (in %)			Average F1 Score Across all Depths (in %)		
	Known miRNA	Novel miRNA	Known piRNA	Known miRNA	Novel miRNA	Known piRNA
miRDeep2	94.74	97.33	-	85.66	85.27	-
miRDeep*	95.67	99.04	-	88.06	95.47	-
mirPRo	78.73	93.04	-	1.45	60.93	-
mirnovo	87.59	91.78	-	60.61	41.95	-
miRge2.0	82.56	0.0	-	25.90	0.0	-
sRNAbench	89.18	0.0	74.25	67.93	0.0	4.34
MiR&moRe2	91.07	92.52	-	74.05	46.76	-
miRPipe	**96.58**	**99.55**	**98.91**	**89.95**	**97.55**	**94.35**

The accuracy and F1-score of known/novel miRNAs and known piRNAs in bold letters represent the outstanding performance of miRPipe pipeline.

#### 3.3.2 Benchmarking of Pipelines on the Identification of Novel miRNAs

Comparison of inter-computational pipelines revealed that miRPipe outperformed all the other computational methods, with an average accuracy of 99.55% and average F1-score of 97.55% across all depths in synthetic data experiments on novel miRNA identification (Table 2; Figure 4, and Supplementary Material S1–S3).

#### 3.3.3 Benchmarking of Pipelines on the Identification of Known piRNAs

We performed a comparative analysis with sRNAtoolbox that uses RNAcentral for piRNA and other snc-RNAs annotations for piRNA identification. Of note, it is the only dedicated computational workflow that allows simultaneous identification of miRNAs and piRNAs. While miRPipe yielded an average accuracy of 98.91% and an average F1-score of 94.35%, sRNAtoolbox yielded an average accuracy of 74.25% and an average F1-score of 4.34% across all depths (Table 2; Figure 4, and Supplementary Material S1–S3).

#### 3.3.4 Overall Benchmarking of all the Pipelines

The overall cumulative performance of all the pipelines is done by considering known/novel miRNAs and piRNAs in the synthetic data experiments and, they are reported in Figure 4. In consistency with the previous results, cumulative performance of miRPipe revealed an average accuracy of 95.22% and an average *F*_1_-score of 94.17% across all depths, a way higher than all tested alternative computational methods (Table 2; Figure 4, and Supplementary Material S1–S3).

#### 3.3.5 Benchmarking of Pipelines on the Identification of Reverse Complement miRNA Sequence as Known miRNAs

**FIGURE 5:**
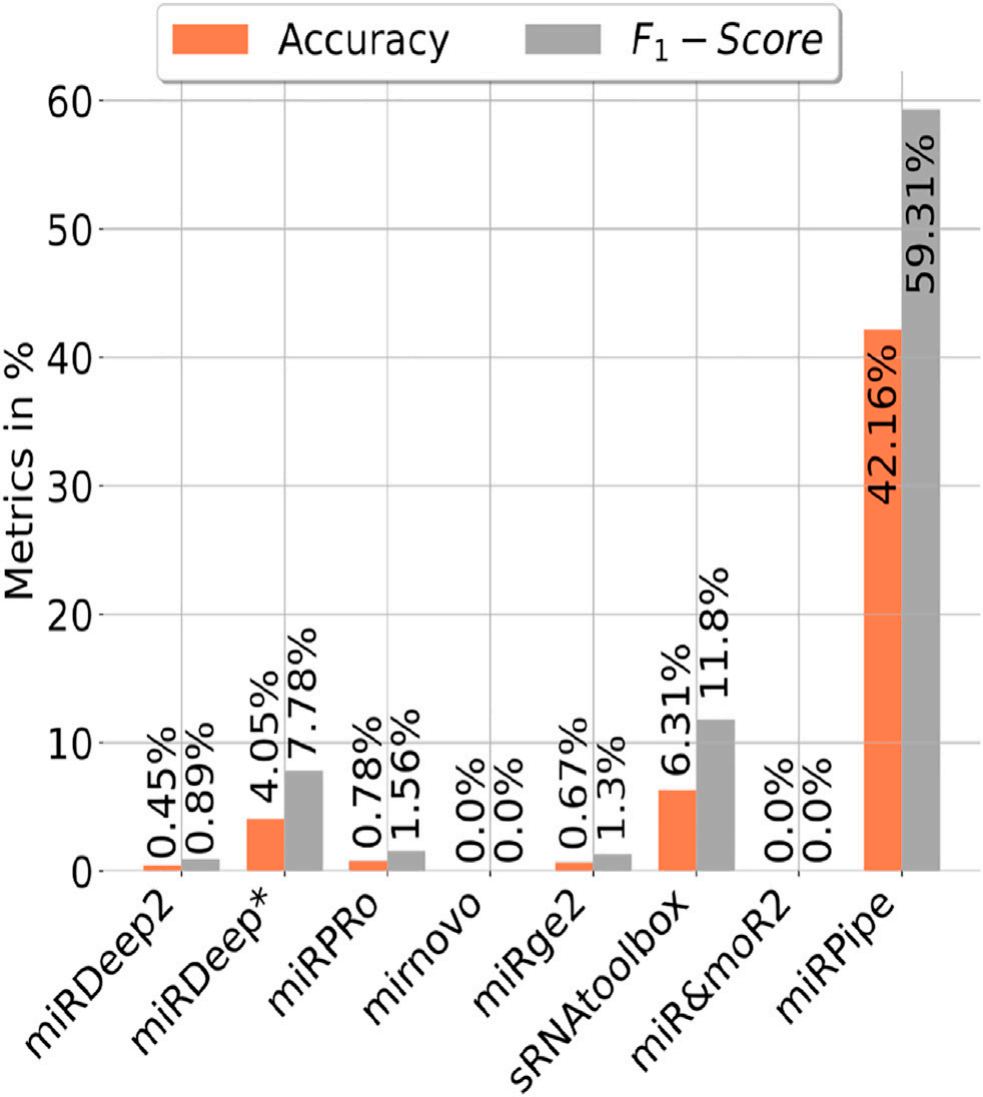
Benchmarking of miRPipe with the seven standard pipelines on the identification of reverse complement miRNA sequence as known miRNA.

We have also benchmarked miRPipe with seven standard pipelines introduced in the recent past for annotation of reverse complement sequence as known miRNAs. As mentioned in Section 2.2 (Synthetic RNA-seq expression dataset used in this study), we have generated the synthetic data for pipeline benchmarking on the annotation of reverse complement sequence using 887 high confidence miRNAs in miRBase database (version 22). The comparative analysis with miRPipe revealed that miRPipe outperformed existing pipelines with an accuracy of 42.16% and an F1-score of 59.31%. We have observed that miRDeep2, miRDeep*, miRPro, mirnovo, miRge2.0, sRNAToolbox, MiR&moRe2, and miRPipe has identified 4, 35, 7, 0, 6, 56, 0, and 374 miRNAs respectively out of 887 high confidence miRNAs in miRBase database (version 22). We have shown the pipeline performance comparison on the identification of the reverse complement miRNA sequence in Figure 5. Although miRPipe has also missed annotating many reverse complement miRNAs, miRPipe has still successfully identified the most number of reverse complement sequences as known miRNAs among all eight pipelines.

### 3.4 miRPipe Validation on Publicly Available Chronic Lymphocytic Leukemia Dataset (GSE123436)

We have validated miRPipe with the publicly available Chronic Lymphocytic Leukemia (CLL) real RNA-Seq expression dataset (GSE123436) for miRNA identification. In the CLL dataset, miRNA profiling of 28 CLL cases and ten age-matched healthy controls were studied to understand the involvement of dysregulated miRNAs in CLL and their impact on clinical outcomes.

#### 3.4.1 Results of all Pipelines on Chronic Lymphocytic Leukemia Dataset (GSE123436)

A total of 31 known miRNAs were found as dysregulated by miRPipe pipeline on CLL real RNA-Seq expression dataset (GSE123436). Out of 31 dysregulated known miRNAs, 24 miRNAs were found as upregulated and 7 miRNAs were downregulated. On the other hand, we have observed that miRDeep2, miRDeep*, miRPro, mirnovo, miRge2.0, sRNAToolbox, and MiR&moRe2 have identified 29, 22, 34, 32, 25, 5, and 5 dysregulated known miRNAs respectively. Further, miRPipe has identified 28 dysregulated piRNAs in CLL real RNA-Seq expression data. Out of 28 dysregulated piRNAs, one piRNA was found to be up-regulated, and the remaining 27 were down-regulated (shown in Table 3). The average percentage of known piRNAs across CLL samples was observed as 5.94% calculated as

$$\%\text{ piRNAs across CLL samples}=\left(\frac{\text{Total no. of sequences annotated as piRNAs}}{\text{Total no. of sequences annotated as piRNAs }+\text{ Total no. of sequences annotated as miRNAs}}\right)*100,$$

**TABLE 3:** Differentially expressed piRNAs in CLL dataset.

S. No.	piRNA	Up/down regulation	Fold change
1	hsa-piR-23019	down	−3.15
2	hsa-piR-23020	down	−3.27
3	hsa-piR-32157	down	−3.21
4	hsa-piR-32158	down	−3.22
5	hsa-piR-32159	down	−3.22
6	hsa-piR-32160	down	−3.22
7	hsa-piR-32161	down	−3.22
8	hsa-piR-32166	down	−3.21
9	hsa-piR-32178	down	−3.21
10	hsa-piR-32181	down	−3.15
11	hsa-piR-32185	down	−3.27
12	hsa-piR-32186	down	−3.22
13	hsa-piR-32194	down	−3.27
14	hsa-piR-32234	down	−3.27
15	hsa-piR-32237	down	−3.27
16	hsa-piR-32838	down	−3.22
17	hsa-piR-32839	down	−3.22
18	hsa-piR-32845	down	−3.18
19	hsa-piR-32852	down	−3.19
20	hsa-piR-32978	down	−3.72
21	hsa-piR-32995	down	−3.72
22	hsa-piR-33013	down	−3.75
23	hsa-piR-32963	up	3.46
24	hsa-piR-32990	down	−1.54
25	hsa-piR-33010	down	−1.54
26	hsa-piR-33053	down	−1.59
27	hsa-piR-32847	down	−1.99
28	hsa-piR-32920	down	−1.16

#### 3.4.2 Literature Validation of all Pipelines on Chronic Lymphocyte Leukemia Dataset (GSE123436)

According to the original publication of this dataset (Kaur et al., 2020), eight miRNAs were found as dysregulated in CLL real RNA-Seq expression dataset. Out of 8 dysregulated miRNAs reported in the original publication, there were five common miRNAs reported by miRPipe. In comparison of dysregulated known miRNAs identified by miRDeep2, miRDeep*, miRPro, mirnovo, miRge2.0, sRNAToolbox, MiR&moRe2 and miRPipe with the literature, 17 out of 29 (58.62%), 18 out of 22 (81.81%), 21 out of 36 (58.33%), 14 out of 25 (56%), 8 out of 25 (32%), 2 out of 5 (40%), 2 out of 5 (40%) and 27 out of 31 (87.09%) miRNAs, respectively, were found to be reported as dysregulated in the literature of CLL. Here, the dysregulated miRNAs identified by miRPipe are found to be reported in multiple CLL-related literature (Balatti et al., 2013; Shen et al., 2014; Ruiz-Lafuente et al., 2015; Balatti et al., 2016; Hu et al., 2018; Yang et al., 2018; Farahat et al., 2019; Gao et al., 2019; Farzadfard et al., 2020; Rahimi et al., 2021; Sitlinger et al., 2021). We have also reported 28 dysregulated piRNAs in CLL which, to the best of our knowledge, no one has reported till date.

#### 3.4.3 Comparison of all Pipeline Results With Reverse Transcription Quantitative Polymerase Chain Reaction on Chronic Lymphocytic Leukemia Dataset (GSE123436)

We next compared the results of dysregulated miRNAs (DEMs) obtained from all the pipelines on CLL real RNA-Seq expression dataset and further validated these findings using semi-quantitative real-time PCR.

miRNA profiling was carried out on treatment naïve 28 CLL cases using the TaqMan Array Human MicroRNA Card A + B v2.0 (Applied Biosystems, CA, United States of America) each of which profiles 380 TaqMan MicroRNA Assays enabling the simultaneous quantitation of 754 (377 + 377) human miRNAs plus 4 endogenous controls. The data was normalized using three endogenous controls U6 SnRNA, RNU48 and RNU44. The results obtained were also validated in additional cohorts of *de novo* CLL patients using the miRCURY LNA™ universal RT microRNA PCR System (Exiqon).The additional data on 89 CLL patients was also generated by our group on the same CLL cohort enrolled in the study as mentioned in Kaur et al. (Kaur et al., 2020). A unique set of 68 DEMs were validated out of 754 miRs tested with TaqMan Array Human MicroRNA Card A + B v2.0 (Applied Biosystems, CA, United States) (Supplementary Material S5).

We have compared the number of DEMs identified by the existing pipelines that matched to the results of RT-qPCR to assess the pipeline performance. In comparison of pipeline performance for miRNA identification, we have observed that each pipeline (including miRPipe) has detected many dysregulated miRNAs (DEMs). However, after comparing them with the results of RT-qPCR, the true DEMs count decreased considerably. This is because it is practically difficult to test and validate all the predicted DEMs in the laboratory for at least three reasons: 1) The assays used for DEM validation may not contain all the predicted DEMs, 2) limitation of sample material available, and 3) it adds a huge cost and extra working hours. Hence, only the topmost or prioritized DEMs are preferably tested and validated. The false-positive miRNAs are the miRNAs that are identified as dysregulated by the pipeline but not validated in RT-qPCR experiments. Similarly, the false-negative miRNAs are the miRNAs that are not identified as dysregulated by the pipeline but are RT-qPCR validated. The % false positives and % false negative of the pipeline are computed as:

$$\%\text{ False Positives}=\left(1-\frac{\text{Number of RT}-\text{qPCR validated miRNAs identified by the pipeline}}{\text{Total number of miRNA identified by the pipeline}}\right)*100,$$

$$\%\text{ False Negatives}=\left(1-\frac{\text{Number of RT}-\text{qPCR validated miRNAs identified by the pipeline}}{\text{Total number of RT}-\text{qPCR validated miRNA}}\right)*100,$$

We combined the RT-qPCR validated miRNAs identified by all eight pipelines to get the total number of RT-qPCR validated miRNAs which give a total of 134 miRNAs. Out of 134 miRNAs, 31 miRNAs were found as RT-qPCR validated. The miRPipe has outperformed all other pipelines with the least false positives and false negatives.In miRPipe, out of 31, 17 miRNAs are found as RT-qPCR validated, giving the least false positives, that is (1–17/31)*100 = 45.16% and least false negatives (1–17/31*100 = 45.16%) among all eight pipelines. The % false positives and % false negatives for the remaining seven pipelines are shown in Table 4 and Supplementary Material S4.

**TABLE 4:** Comparison of pipeline performance in CLL real RNA-Seq expression dataset.

S. No.	Pipeline	No. of Dysregulated miRNA Identified by Pipeline	Number of miRNAs Validated with RT-qPCR Results	% False Positive	% False Negative
1	miRDeep2	29	9	68.96	70.96
2	miRDeep*	22	10	54.54	67.74
3	miRPro	34	12	64.70	61.29
4	mirnovo	32	6	81.25	80.64
5	miRge2.0	25	4	84	87.09
6	sRNAToolbox	5	1	80	96.77
7	MiR&moRe2	5	1	80	96.77
**8**	**miRPipe**	**31**	**17**	**45.16**	**45.16**

The bold letters represent the number of miRNAs identified, number of RT-qPCR validated mIRNAs, % False Positives, and % False Negatives reported by miRPipe pipeline.

### 3.5 miRPipe Validation on Publicly Available Lung *Cancer* Dataset (GSE37764)

We have validated miRPipe with the publicly available lung cancer dataset (GSE37764) for piRNA identification. In the lung cancer dataset, the role of dysregulated miRNAs and piRNAs in the non-smoker female lung cancer patients were studied. Among eight pipelines used for benchmarking, only miRPipe and sRNAToolbox identify piRNAs. According to the original publication of this dataset (Nogueira Jorge et al., 2017), no piRNAs were found to be dysregulated in RNA-Seq samples of non-smoker females. However, a total of 18 and 20 dysregulated piRNAs were identified by the miRPipe and sRNAToolbox, respectively (Supplementary Material S7). There was no common piRNA detected by the two pipelines. Out of the 18 piRNAs (identified by miRPipe), 6 piRNAs (33.33%) were found to be reported as dysregulated in lung adenocarcinoma in the literature (Mei et al., 2015). On the contrary, none of the piRNAs identified by sRNAToolbox were found to be reported in the literature.

### 3.6 miRPipe Validation on Publicly Available Breast *Cancer* Dataset (GSE171282)

We have also validated the miRNA identification pipeline in miRPipe with a publicly available breast cancer dataset (GSE171282). In (Lin et al., 2021), 11 dysregulated miRNAs were identified to understand their involvement in the effects of anesthetics on breast cancer cells. We have observed that miRDeep2, miRDeep*, miRPro, mirnovo, miRge2.0, sRNAToolbox, MiR&moRe2, and miRPipe have identified 22, 8, 31, 29, 34, 14, 42, and 21 known dysregulated miRNAs respectively (Supplementary Material S8). In comparing with the literature reported miRNA 9 out of 22 (40.90%), 7 out of 8 (87.5%), 10 out of 31 (32.25%), 8 out of 29 (27.58%), 19 out of 34 (55.88%), 12 out of 14 (85.71%), 23 out of 42 (54.76%), and 19 out of 21 (90.47%) miRNAs were found to be reported as dysregulated in the literature of breast cancer. Here, the dysregulated miRNAs identified by miRPipe are found to be reported in multiple breast cancer-related research papers (Shi Y. et al., 2015; Krishnan et al., 2015; Hannafon et al., 2016; Tan et al., 2016; Li et al., 2017; Reza et al., 2017; Schultz et al., 2017; Sripada et al., 2017; Xia et al., 2017; Chen et al., 2018; Lagendijk et al., 2018; Lai et al., 2019; Li et al., 2019; Sun et al., 2019; Xie et al., 2019; Mahlab-Aviv et al., 2020; Zhang et al., 2020; Ghafouri-Fard et al., 2021; Shen et al., 2021; Yang et al., 2021; Zellinger et al., 2021; Park et al., 2022). Only 6 out of 11 (54.54%) miRNAs reported in the original publication of this dataset (Lin et al., 2021) were found to be reported as dysregulated in the literature. Of the pipelines compared, miRPipe and MiR&moRe2 reported miRNAs matched most with the literature (19 and 23, respectively), although miRPipe has the least number of FPs because of the 21 reported by miRPipe, 19 matched with the literature.

## 4 Discussion

In this work, we have benchmarked our pipeline, miRPipe, with seven recent pipelines (miRDeep2, miRDeep*, mirPRo, mirnovo, miRge2.0, sRNAtoolbox, and MiR&moRe2) using a newly developed synthetic RNA-sequence simulator, miRSim tool that generates FASTQ file with known fraction of altered/unaltered known/novel miRNAs and piRNAs, and help evaluate pipelines on identifying true positives and rejecting false miRNA/piRNA reads looking similar to known miRNAs/piRNAs.

### 4.1 Difference in miRDeep2, miRDeep* and miRPipe

We have added the following methods in miRPipe to make it better then miRDeep2 and miRDeep*:

1) *Novel Seed-based clustering*: Both, miRDeep* and miRDeep2 do not report the known miRNA paralogues and yield many false positives and false negatives, which reduce their accuracy and F1 score. In Step-3 of the miRPipe workflow (that is, by miRDeep*), there can be many novel miRNA sequences that are not assigned to their correct miRNA family, or in other words, they are not detected properly. For example, some paralogues of known miRNAs are declared as novel miRNAs by the sequence aligner in Step-3 of miRPipe (miRDeep*), although they should have been assigned to their respective known miRNA families. miRPipe clusters such miRNAs declared as novel in Step-3 of miRPipe using novel seed-based clustering (Step-6). In Step-6, miRPipe identifies novel miRNAs and known miRNA paralogues by comparing the seed, xseed sequence (other than the seed sequence), and their genomic locations. Similarly, Step-6 of the miRPipe workflow also combines novel miRNAs sharing the same seed sequence as that of a known miRNA (or another novel miRNA), maximum of two alterations in xseed sequence and similar genomic location through seed-based clustering. After Step-6, miRPipe eventually yields uniquely identified novel miRs and their paralogues. This step helps miRPipe to yield the least false positives and false negatives. For example, let us consider a sequence “tcc​ctg​tcc​tcc​agg​agc​tc” that is identified as novel miRNA (say novelMir-1) in Step-3 of the miRPipe workflow. The novelMir-1 has an identical seed as that of hsa-mir-339, has more than 2 nt alteration in the xseed region, and is mapped at a genomic location other than that of hsa-mir-339. Therefore, novelMir-1 should be called a paralogue of hsa-mir-339 and should be labeled as hsa-mir-339_1. Thus, novelMir-1 naming leads to a false positive to the novel miRNA class and a false negative for the known miRNA class. In miRPipe pipeline, by assigning the correct class to novelMir-1 as hsa-mir-339_1, both the false positive and false negative would be reduced.
2) *Identification of reverse complement miRNAs as known miRNA using DASHR blast search*: miRPipe checks whether the miRNAs identified as a novel miRNA in Step-3 of the miRPipe pipeline are indeed novel. In Step-3 of the miRPipe workflow, there can be some sequences that are annotated as novel miRNAs, whose annotation is missed due to it being present as a reverse complement sequence in the fastq file. miRDeep2 fails to identify the reverse complement sequence as known miRNA. Out of 887 high confidence known miRNAs, miRDeep2 has correctly annotate only 4 reverse complement sequence as known miRNAs. Moreover, miRDeep* can annotate only those reverse complement sequence as a known miRNA that are already annotated in the miRBase database, regardless of mapping strand of the reverse complement sequence with the human genome. For example, the miRNAs hsa-mir-3529–5p (agg​tag​act​ggg​att​tgt​tgt​t) and hsa-mir-7–2 (aac​aac​aaa​tcc​cag​tct​acc​t) are reverse complementary pair. The reverse complement of hsa-mir-3529–5p (or hsa-mir-7–2) will be mapped to hsa-mir-7–2 (or hsa-mir-3529–5p) in the opposite strand. Similarly, for the reverse complimentary pair hsa-mir-103a-3p (agc​agc​att​gta​cag​ggc​tat​ga) and hsa-mir-103b-1 (tca​tag​ccc​tgt​aca​atg​ctg​ct), the reverse complement of hsa-mir-103a-3p (or hsa-mir-103b-1) will be mapped to hsa-mir-103b-1 or (hsa-mir-103a-3p) in the same strand. Out of 887 high confidence known miRNAs, miRDeep* has correctly annotate only 35 reverse complement sequence as known miRNAs. However, in many cases, due to different mapping strand and precursor sequence of the reverse complement sequence with the respective mapping strand and precursor sequence of that known miRNA, miRDeep* failed to annotate the reverse complement sequence to its true known miRNA and annotated them as novel miRNAs. Due to lack of correct annotation of reverse complement sequences as known miRNAs, both miRDeep2 and miRDeep* yield many false positives and false negatives. On the other hand, miRPipe correctly annotate the reverse complement sequence to its true known miRNA in the Step-5 of the workflow (DASHR blast search). For example of a sequence “cta​cag​agg​cga​cat​ggg​ggt​ca” (say mir-1) which is the reverse complement of hsa-mir-6859–3p (tga​ccc​cca​tgt​cgc​ctc​tgt​ag). The sequence of mir-1 is mapped at the genomic location chr1:17,369–17,391 (chromosome^.^number:chromosome^.^start, chromosome^.^end) which is the same as the genomic location of hsa-mir-6859–3p reported in miRBase database. The mapping strand of mir-1 is opposite with the respective strand of hsa-mir-6859–3p. The precursor sequence generated by miRDeep* for mir-1 is the reverse complement with the respective precursor sequence of hsa-mir-6859 in miRBase. Hence, miRDeep* will annotate mir-1 as novel miRNA, while miRPipe will correctly annotate mir-1 to hsa-mir-6859 in Step-5 of the workflow (DASHR blast search).
3) *Identification of piRNA*: Unlike most of the bioinformatics pipelines that either identify miRNAs or piRNAs, miRPipe also identifies piRNAs along with the miRNAs from the RNA-Seq data.
4) *Customized reference genome*: miRPipe allows users to choose the reference genome hg19/hg38) and miRBase version (version 19/20/21/22) as per the requirement. The sequence aligner used in miRPipe uses the miRBase database for sequence annotation. If required, a user can add another database for miRNA annotation. For example, MirGeneDB can be used instead of miRBase, and accordingly, the sequences can be annotated according to this database. If a user replaces the miRBase annotation files with that of mirGeneDB, then miRPipe will annotate the miRNA according to the MirGeneDB database.
5) *Batch-mode operation*: Since miRDeep* is a single-threaded memory-intensive sequence aligner, the sequential operation increases the time taken by the pipeline when data of multiple subjects is required to be processed. On the other hand, miRPipe allows the execution of sequence alignment in the batch mode for multiple subjects’ data analysis and therefore, has significantly reduced execution time in the downstream analysis. In order to provide operational flexibility in miRPipe, a user can control whether to run a job in the sequential mode (one subject’s file or one sample file at a time) or in the batch mode (multiple subjects’ files or multiple samples’ files). In sequential mode, miRPipe will align one file at a time. Similarly, in batch mode, the entire dataset consisting of multiple files is divided into several small batches. All these batches are processed parallelly on dedicated (individual) CPU threads. Further, the user can also control the number of threads and memory allocation per thread (as per the system hardware RAM limits).This operation is faster and less time-consuming than the sequential operation for a big dataset.
6) *Cohort analysis and identification of dysregulated miRNAs*: miRPipe can perform cohort analysis (dataset containing multiple samples) and report the dysregulated known miRNAs, novel miRNAs, and known piRNAs via the statistical test of DESeq2. For cohort analysis, miRPipe can split the cohort into multiple batches and process each batch on a dedicated thread parallely, and then use DESeq2 to report dysregulated miRNAs or piRNAs. On the contrary, since miRDeep* can process only one sample at a time, it does not report the dysregulated miRNAs or dysregulated piRNAs, but can only detect miRNAs present in the fastq file of a subject.
7) *Synthetic Data Generator (miRSim)*: We have also developed the miRSim tool to generate synthetic data for the extensive benchmarking of different pipelines.
8) Both miRSim and miRPipe are open-source and available publicly in an interactive jupyter notebook at the GitHub repositories.
9) *Selective pipeline execution*: We have developed miRPipe in an interactive jupyter notebook. The miRPipe pipeline is developed in such a way that both piRNA and miRNA pipeline can run together. If a user wants to run only one pipeline at a time, that can be done easily in the jupyter notebook.

### 4.2 Comparison of all Pipelines on Known miRNA Identification

Of the existing pipelines, miRDeep2 identifies miRNAs by hierarchical sequence alignment followed by RNA secondary structure prediction of potential precursors and estimation of the performance statistics of all potential precursors to filter false positives. However, it allows mismatches of 1 to 2 nt in the reads while matching the corresponding sequence to those of known miRNAs introducing false positives. In addition, if there is a reverse complement of a known miRNA sequence, it either rejects it or annotates it as novel miRNA. On the other hand, miRdeep* follows the same methodology as miRDeep2, except that it incorporates an improved strategy for miRNA precursor sequence identification and additional isomiR detection capacity. Further, it does not allow any mismatch with known miRNAs, unlike miRdeep2, reducing the false positives. mirPRo also follows the same methodology of miRDeep2 except, that it imposes a stringent condition, wherein only perfectly mapped reads are allowed for known miRNA prediction. mirPRo also includes isomiR detection. mirPRo pipeline does not report the paralogues of known or novel miRNAs. mirPRo uses a Novoalign sequence aligner for the identification of known miRNAs, allowing a maximum 2 nt mismatch or three indels in one opening gap. This could be the reason for more false positives with mirPRo.

We observed that six out of eight pipelines performed well on known miRNA. Of these, miRPipe, miRDeep2, and miRDeep* performed best, while mirnovo, miRge2.0, and sRNAtoolbox yielded average performance, while mirPRo comparatively underperformed. The performance of miRDeep2 was close to miRDeep* except for a few miRNAs, whose precursor were inconsistent for dicer processing. On the other hand, the miRDeep* tool has an improved precursor excision strategy over miRDeep2, leading to better performance on known miRNA identification. We have observed that the average accuracy and average F1-score of miRDeep2 and miRDeep* across all depth for known miRNA identification was 94.74%, 85.66% and 95.67%, 88.06%, respectively while miRPipe has an average accuracy and average F1-score of 96.58 and 89.95% (Table 2). The improvement in the miRPipe performance on the identification of known miRNAs was due to DASHR blast search and seed-based clustering method.

### 4.3 Comparison of all Pipelines on Novel miRNA Identification

For novel miRNA identification, miRDeep2, miRDeep*, mirPRo, MiR&moRe2, and miRPipe use the hybrid approach that includes both genomic features and hairpin structural features. A sequence has to pass through 6 conditions to be annotated as novel miRNAs, such as 1. Position of potential mature sequence to potential hairpin sequence, 2. Potential star sequence, 3. Potential loop sequence, 4. Number of base pairs between mature and star sequence, 5. Percentages of reads aligned to the location of mature miRNA for proper dicer processing (at least 90% read should be aligned) and, 6. Log-odds probability score for potential mature miRNA. These six conditions are used to rigorously scan the precursor sequence to identify a read as a novel miRNA. miRDeep* additionally employs the improved precursor excision strategy compared to miRDeep2, which leads to better performance. mirPRo has improved performance on novel miRNA than known miRNA detection. It also shows better performance on novel miRNA identification compared to miRge2.0, sRNAtoolbox, and MiR&moRe2 because mirPRo follows the same six conditions and additionally allows a maximum mismatch of 1 nt. It considers mapped read lengths between 18 and 25 nt and the fold-change criterion (that is, keep only mapped reads with the highest read stack with at least two-fold change compared to the second-highest read stack) to reduce the false positives. Since, miRPipe is an improvisation for reducing false positives and false negatives by incorporating DASHR blast search and seed-based clustering on novel miRNAs sequences, it yields better results than these tools and other pipelines. Of note, miRPipe has the lowest false positives and false negatives in comparison to other pipelines.

sRNAToolbox imposes stringent conditions for novel miRNA prediction such as within-cluster ratio, 5’ fluctuation, minimum number of hairpin bindings, the minimum number of mature bindings, length intervals, and minimum reads. The threshold for each feature is derived from the same machine-learning model training dataset used in miRAnalyzer (Hackenberg et al., 2011). We have observed that no novel miRNA was identified in miRSim simulated synthetic data due to the sRNAToolbox stringent conditions. The sRNAToolbox has also not identified any novel miRNAs in the synthetic data experiment on the identification of reverse complement sequence as known miRNA. Moreover, sRNAToolbox has reported only three, one, and three novel miRNAs in the CLL dataset (GSE123436), lung cancer dataset (GSE37764), and breast cancer dataset (GSE171282) dataset, respectively. None of the novel miRNA were found as dysregulated in differential expression analysis in any of the datasets. Similarly, miRge2.0 utilizes an SVM machine-learning model that uses 22 structural and compositional features for novel miRNA predictions. The SVM model has been trained on 17 tissues of the human and mouse datasets. Due to these stringent conditions, miRge2.0 did not report any novel miRNAs in synthetic data benchmarking experiments. Moreover, the miRge2.0 pipeline identified 18, zero, and zero novel miRNAs in the CLL dataset (GSE123436), lung cancer dataset (GSE37764), and breast cancer dataset (GSE171282) dataset, respectively. None of identified novel miRNA were found as dysregulated in differential expression analysis in any of the datasets. This could be due to the lack of generalizability of the SVM model trained by miRge2.0 leading to such high false negatives.

Similarly, the mirnovo pipeline uses machine learning (random forest model) with 12 coverage profile features, 12 sequence complexity, and nine genomic features hairpin structural features for novel miRNA identification. It provides not only novel miRNAs but also other non-coding RNAs such as tRNA or rRNAs. It is also observed to have high false negatives. All the three above (sRNAToolbox, miRge2.0, and mirnovo) are simple methods that do not impose many stringent conditions for detecting novel miRNAs and hence, lead to many false positives.

MiR&moRe2 identifies loop-RNAs, microRNA-offset RNAs (moRNAs), and novel miRNAs with the precursor excision methodology similar to miRDeep2, except that the candidate precursor sequences are extended to 30 nt on both upstream and downstream for the identification of the possible moRNAs. It also checks for the sequences that are aligned in the offset region or loop region of the miRNAs hairpin and can be annotated as microRNA-offset RNAs (moRNAs) and loop-RNAs. The miRNAs sequences that are neither moRNAs nor loop-RNAs and located in the close proximity to the mature sequence of the hairpin precursor are considered as novel miRNAs. MiR&moRe2 lacks the identification paralogues and have many false negatives due to an inefficient precursor excision strategy. miRPipe addresses the issues of identification of paralogues, functional annotation of novel miRNAs, utilizing both the genomic and precursor features, and hence, outperform all the other pipelines.

### 4.4 Comparison of all Pipelines on Known piRNA Identification

Among all these pipelines, only miRPipe and sRNAtoolbox identify piRNAs and hence, reported these in the synthetic data experiments. We observed average accuracy and a low *F*_1_-score for piRNA identification in the sRNAtoolbox due to high false negatives. In miRPipe, the stringent condition of zero nucleotide mismatch in the seed region and no reverse complement alignment helped in reducing the false positives during piRNA identification. Compared to other pipelines, sRNAtoolbox also reports other non-coding RNAs (long non-coding RNAs, piRNAs etc.) using blast search for all unmapped/unassigned reads to several remote databases hosted at NCBI (such as GenBank, EMBL etc.) with the help of several helper tools in sRNAbench such as Ensembl Parser, NCBI Parser, RNA central parser, and Genomic tRNA database parser.

### 4.5 General Remarks

It is possible that the combination of different methods can improve the results. The combination of multiple methods can be either the consensus of results of all methods or the union of results of all methods. If we consider the consensus results, it is possible to reduce false positives. However, it may lead to high false negatives because of the methodological differences of pipelines that impact miRNA identification. Similarly considering the union results, it may lead to high false positives, which is also not good. We believe that miRPipe addresses this issue because miRPipe is an end-to-end unified workflow that can report all important miRNAs/piRNAs in one go with the least false positives or false negatives, as shown in the benchmarking results.

We have validated miRPipe using miRSim simulated synthetic data with ground truth and three publically available real RNA-Seq expression datasets (GSE123436, GSE37764, GSE171282).The bioinformatics pipeline can also be validated using some publicly available sequencing data with added synthetic microRNAs, usually using an equimolar mixture of 962 synthetic microRNAs miRXplore universal Reference from Miltenyi (Heinicke et al., 2020). Further, miRPipe or any other bioinformatics pipeline can also be tested on the comprehensive atlas of the human transcriptome from “The RNA Atlas expands the catalog of human non-coding RNAs.” (Lorenzi et al., 2021), which includes small, polyA RNA as well as total RNA from 300 human tissues and cell lines. Since miRPipe is an open-source bioinformatics pipeline, any future researcher can test the pipeline on these datasets. As of now, miRPipe pipeline is applicable only for human genomics data. For other genomes, users need to replace the human genome reference index with the corresponding non-human genome reference index and also link the corresponding sequence annotation database. After replacing the reference index and annotation files, miRPipe can be used for the non-human genome as the core algorithm will remain the same.

## 5 Conclusion

The synthetic data experiment validation and benchmarking strategy, along with the validation on real RNA-Seq expression data, establishes miRPipe as a robust, reliable, and reproducible pipeline for the detection of known/novel miRNAs, paralogues, and piRNAs from the RNA-Seq data. miRPipe outperforms recent state-of-the-art pipelines. miRPipe can jointly identify miRNAs and piRNAs and carries out parallel batch processing for the efficient utilization of the computational resources. The IPython notebook for bioinformatics pipeline and containerization of tools makes its configuration and deployment easy with minimum effort.

## Data Availability

All the source codes necessary to reproduce the results given in Figure 3A–L and the Supplementary Tables S1-3 are available in the github (https://github.com/vivekruhela/miRPipe) for the synthetic data. The synthetic fastq data files are also available at the same repository. The open-source synthetic data simulator tool miRSim can be access from github(https://github .com/vivekruhela/miRSim). The RNA-Seq CLL real RNA-Seq expression data can be accessed from the repository GSE123436 (https://www.ncbi.nlm.nih.gov/geo/query/acc.cgi?acc= GSE123436). Similarly, lung cancer and breast cancer real RNA-Seq expression data can be accessed from the repository GSE37764 (https://www.ncbi.nlm.nih.gov/geo/query/acc.cgi?acc=GSE37764) and GSE171282 (https://www.ncbi.nlm.nih.gov/geo/query/acc.cgi?acc=GSE171282) respectively.
