# Supplementary material for: A Unified Computational Framework for a Robust, Reliable, and Reproducible Identification of Novel miRNAs From the RNA Sequencing Data
Source: Front Bioinform. 2022 Jul 8;2:842051. doi: 10.3389/fbinf.2022.842051 (PMC9580950; doi:10.3389/fbinf.2022.842051)
Supplement: Supplementary file 4 [file DataSheet5.docx]

miRNA profiling was carried out on 28 CLL cases using the TaqMan Array Human MicroRNA Card A+ B v2.0 (Applied Biosystems, CA, USA) each of which profiles 380 TaqMan MicroRNA Assays enabling the simultaneous quantitation of 754 (377+377) human miRNAs plus 4 endogenous controls. The data was normalised using three endogenous controls U6 SnRNA, RNU48 and RNU44. The results obtained were also validated in additional cohorts of de novo CLL patients using the miRCURY LNA™ Universal RT microRNA PCR System (Exiqon). Twenty six miRNAs including **miR-15a-5p**, **miR-23a-5p**, **miR-138-5p**, **miR-143-3p, miR-145-3p**, hsa-**miR-193a-5p**, **miR-223-3p, miR-330-5p**, **miR-338-3p, miR-548a-5p**, **miR-551a, miR-582-5p, miR-582-3p, miR-597-5p, miR-601, miR-645, miR-661, miR-944 (n=69)** and; mir-30a, mir-1295a, mir-155, mir-4525a, mir-423, mir-486, mir-let-7e and mir-744 (n=89) were assayed. Fold change was calculated using SNORD44 and SNORD48 as endogenous controls. The data were collected using Quantstudio12KFlex software v1.2.2 (Applied Biosystems) and processed using the Expression suite software v1.0.4 (Thermofisher Scientific).

***CLL Vs Normal:***

**Identification of DEMs in CLL by TLDA**

A unique set of 68 DEMs were validated out of 754 tested with TLDA approach in CLL as compared to healthy controls (Table 5).

**Table 5: Significant differentially expressed miRNAs in CLL identified by TLDA**

| **Sr No** | **Target Name** | **RQ (Fold Change) CLL Vs Normal** | **P-Value** |
| --- | --- | --- | --- |
|  | has-miR-1305 | 0.655 | 0.043 |
|  | hsa-miR-1 | 0.099 | 0.007 |
|  | hsa-miR-103 | 0.404 | 0.000 |
|  | hsa-miR-106a | 0.423 | 0.000 |
|  | hsa-miR-10b | 0.070 | 0.045 |
|  | hsa-miR-1225-3P | 3.578 | 0.031 |
|  | hsa-miR-1226 | 0.340 | 0.000 |
|  | hsa-miR-1254 | 0.306 | 0.003 |
|  | hsa-miR-126 | 0.048 | 0.013 |
|  | hsa-miR-1282 | 0.356 | 0.042 |
|  | hsa-miR-1300 | 1.135 | 0.037 |
|  | hsa-miR-1301 | 0.060 | 0.000 |
|  | hsa-miR-130a | 0.054 | 0.009 |
|  | hsa-miR-130b | 0.313 | 0.022 |
|  | hsa-miR-135b | 2.879 | 0.014 |
|  | hsa-miR-143 | 0.282 | 0.032 |
|  | hsa-miR-145 | 0.213 | 0.000 |
|  | hsa-miR-146a | 0.280 | 0.004 |
|  | hsa-miR-148b | 0.469 | 0.004 |
|  | hsa-miR-149 | 0.476 | 0.009 |
|  | hsa-miR-150 | 1.810 | 0.007 |
|  | hsa-miR-151-5P | 0.098 | 0.001 |
|  | hsa-miR-155-5p | 4.038 | 0.000 |
|  | hsa-miR-15b-5p | 0.274 | 0.001 |
|  | hsa-miR-15b-3p | 0.416 | 0.000 |
|  | hsa-miR-181a | 0.179 | 0.018 |
|  | hsa-miR-184 | 3.561 | 0.016 |
|  | hsa-miR-18a | 0.300 | 0.000 |
|  | hsa-miR-199a | 0.074 | 0.044 |
|  | hsa-miR-21 | 2.211 | 0.016 |
|  | hsa-miR-218 | 4.798 | 0.000 |
|  | hsa-miR-221 | 0.313 | 0.033 |
|  | hsa-miR-222 | 0.403 | 0.005 |
|  | hsa-miR-24-2 | 0.311 | 0.000 |
|  | hsa-miR-296-3p | 1.495 | 0.001 |
|  | hsa-miR-29b-2 | 0.350 | 0.015 |
|  | hsa-miR-301 | 0.350 | 0.001 |
|  | hsa-miR-320B | 0.368 | 0.000 |
|  | hsa-miR-330-5p | 1.968 | 0.000 |
|  | hsa-miR-339-5p | 0.299 | 0.000 |
|  | hsa-miR-34a# | 1.367 | 0.013 |
|  | hsa-miR-34a | 2.002 | 0.002 |
|  | hsa-miR-361-3p | 0.456 | 0.000 |
|  | hsa-miR-380-5p | 0.073 | 0.001 |
|  | hsa-miR-424 | 1.134 | 0.044 |
|  | hsa-miR-429 | 0.266 | 0.000 |
|  | hsa-miR-450b | 1.403 | 0.008 |
|  | hsa-miR-454 | 0.546 | 0.022 |
|  | hsa-miR-455-3p | 4.832 | 0.045 |
|  | hsa-miR-486-3p | 1.392 | 0.001 |
|  | hsa-miR-511 | 19.150 | 0.007 |
|  | hsa-miR-513-5p | 4.333 | 0.039 |
|  | hsa-miR-517a | 1.236 | 0.006 |
|  | hsa-miR-517b | 5.407 | 0.002 |
|  | hsa-miR-520D | 0.333 | 0.044 |
|  | hsa-miR-522 | 1.744 | 0.041 |
|  | hsa-miR-542-3p | 1.302 | 0.021 |
|  | hsa-miR-548b | 1.244 | 0.020 |
|  | hsa-miR-551b | 0.192 | 0.000 |
|  | hsa-miR-625#- | 0.142 | 0.000 |
|  | hsa-miR-625 | 0.190 | 0.000 |
|  | hsa-miR-638 | 0.626 | 0.036 |
|  | hsa-miR-645 | 0.391 | 0.005 |
|  | hsa-miR-656 | 0.055 | 0.048 |
|  | hsa-miR-7-2# | 0.482 | 0.000 |
|  | hsa-miR-874 | 2.021 | 0.000 |
|  | hsa-miR-942 | 0.438 | 0.000 |
|  | mmu-miR-451 | 2.514 | 0.007 |

**Following miRs were validated using RT-qPCR on a cohort of 69 patients:**

| **Sr No** | **Target Name** | **RQ (Fold Change) CLL Vs Normal** | **P-Value** |
| --- | --- | --- | --- |
|  | miR-138-5p | 0.005 | 0.030 |
|  | miR-145-3p | 0.133 | 0.049 |
|  | miR-15a-5p | 0.078 | 0.049 |
|  | miR-223-3p | 0.201 | 0.049 |
|  | miR-582-5p | 0.209 | 0.0057 |
|  | miR-597-5p | 0.739 | 0.0549 |
|  | miR-582-3p | 0.153 | 0.035 |

For other miRs, the fold change are as follows miR-145-3p (FC=0.138, p=0.067), miR-193a-5p (FC=0.309, p=0.078), miR-330-5p (FC=0.571, p=0.136), miR-338-3p (FC=0.454, p=0.119), miR-548a-5p (FC=0.323, p=0.067) and miR-551a (FC=0.619, p=0.421).

**8 miRs validated on a cohort of 89 patients (published in BCJ paper):**

| **Sr No** | **Target Name** | **RQ (Fold Change) CLL Vs Normal** | **P-Value** |
| --- | --- | --- | --- |
|  | miR-1295a | 8.28 | 0.000932 |
|  | miR-4524a | 7.39 | 0.00299 |
|  | miR-155 | 2.06 | 0.00101 |
|  | miR-30a | -4.19 | 1.236E-08 |
|  | let-7e | -3.59 | 0.0293 |
|  | miR-744 | -2.63 | 0.0293 |
|  | miR-486* | -1.54 | 0.0155 |
|  | miR-423 | -1.41 | 0.0155 |
